# *IL12B* rs3213094 as a Predictor of Early Response to Biologic Therapy in Psoriasis: A Real-World Study in a Romanian Cohort

**DOI:** 10.3390/medicina62061041

**Published:** 2026-05-28

**Authors:** Alessandra-Madalina Matei-Man, Ildiko-Orsolya Gaal, Andreea Catana, Stefan Vesa, Simona Senila, Elisabeta Candrea, Meda Orasan, Alexandra Puskas, Ana Calina Man, Teodora Mocan

**Affiliations:** 1 Dermatology Department, Faculty of Medicine, “Iuliu Haţieganu” University of Medicine and Pharmacy, 400126 Cluj-Napoca, Romania; madalina.man@outlook.com (A.-M.M.-M.); simonasenila@yahoo.com (S.S.); elisa_candrea@yahoo.com (E.C.); 2 Department of Medical Genetics, “Iuliu Haţieganu” University of Medicine and Pharmacy, Pasteur Street 6, 400349 Cluj-Napoca, Romania; orsigaal92@gmail.com (I.-O.G.); catanaandreea@gmail.com (A.C.); 3 Department 2 Functional Sciences, Discipline of Pharmacology, Toxicology and Clinical Pharmacology, Faculty of Medicine, “Iuliu Haţieganu” University of Medicine and Pharmacy, 400337 Cluj-Napoca, Romania; 4 Physiopathology Department, Faculty of Medicine, “Iuliu Haţieganu” University of Medicine and Pharmacy, 400126 Cluj-Napoca, Romania; meda2002m@yahoo.com; 5 Independent Researcher, 1021 Budapest, Hungary; dr.alexandradana@gmail.com; 6 Life Sciences, Biotechnology Department, Harvard Extension School, Cambridge, MA 02138, USA; anm7964@harvard.edu; 7 Physiology Department, Faculty of Medicine, “Iuliu Haţieganu” University of Medicine and Pharmacy, 400126 Cluj-Napoca, Romania; teodora_mocan@yahoo.com

**Keywords:** psoriasis, *IL12B*, rs3213094, *IL23R*, rs11209026, genetic polymorphism

## Abstract

*Background and Objectives*: Psoriasis is a chronic immune-mediated inflammatory disease characterized by heterogeneous clinical presentation and variable response to biologic therapy. Genetic variation within the IL-23/Th17 inflammatory pathway may influence treatment outcomes. This study evaluated the association between *IL12B* rs3213094 and *IL23R* rs11209026 single-nucleotide polymorphisms (SNPs) and response to biologic therapy in patients with moderate-to-severe psoriasis. *Materials and Methods*: We conducted a multicenter observational study including 92 Romanian patients with moderate-to-severe psoriasis vulgaris receiving their first biologic therapy (anti-TNF, anti-IL-17, or anti-IL-23 monoclonal antibodies). Clinical response was assessed using the Psoriasis Area and Severity Index (PASI) at baseline and weeks 12, 24, 36, and 48. Early response was defined as achieving PASI75 at week 12. Patient-reported disease impact was assessed using the Dermatology Life Quality Index (DLQI) at the same time points. Genotyping of *IL12B* rs3213094 and *IL23R* rs11209026 was performed using TaqMan assays. Longitudinal PASI dynamics were analyzed using repeated-measures ANOVA, while multivariable logistic regression was used to identify independent predictors of PASI75 at week 12. *Results*: A significant reduction in PASI scores over time was observed (*p* < 0.001). The *IL12B* rs3213094 genotype was associated with differences in early response kinetics, with T-allele carriers showing significantly greater PASI improvement at week 12 compared with CC homozygotes (90.0% vs. 65.7%, *p* = 0.003). This effect was limited to early treatment and attenuated at later time points. In multivariable analysis, the *IL12B* rs3213094 CT + TT genotype was independently associated with PASI75 achievement at week 12 (OR = 4.285, 95% CI 1.500–12.239, *p* = 0.007). Treatment with anti-IL-17 agents was also an independent predictor of early response (OR = 3.946, 95% CI 1.416–10.998, *p* = 0.009). No significant association was observed between *IL23R* rs11209026 and treatment response. DLQI scores improved significantly over time (*p* < 0.001), without genotype-dependent differences. *Conclusions*: *IL12B* rs3213094 SNP is significantly associated with early biologic treatment response in psoriasis, supporting its potential role as a pharmacogenetic biomarker of treatment responsiveness. These findings may inform the integration of genetic markers into personalized therapeutic strategies, particularly in underrepresented populations such as those from Eastern Europe. Further studies in larger cohorts are warranted to validate these results.

## 1. Introduction

Psoriasis is a chronic, immune-mediated inflammatory skin disease, with a global prevalence of 2–4%, representing a serious public health burden [1]. Psoriasis not only affects the skin. Nowadays, it is widely recognized as a multisystem disorder associated with arthritis, cardiovascular disease, metabolic syndrome, and even psychiatric comorbidities, all of which contribute to reduced quality of life [2]. The high socioeconomic impact of psoriasis is reflected in millions of incident cases reported annually [3].

The pathogenesis of psoriasis comprises complex interactions among genetic predisposition, environmental factors, and immune dysregulation [4]. Central to disease pathogenesis is the IL23/Th17 axis, which drives inflammation and keratinocyte hyperproliferation [5]. The role of immune-mediated mechanisms in disease initiation and progression is underscored by genome-wide association studies (GWASs), which have revealed numerous susceptibility loci, including NF-κB (a master regulator of inflammation) [4] and several HLA-related loci, which represent the strongest genetic contributors to psoriasis susceptibility [6].

Genetic factors are estimated to account for approximately 60–70% of psoriasis susceptibility. However, environmental factors such as smoking, obesity, infections, and psychological stress play a critical role in triggering and modulating disease activity. Consequently, marked heterogeneity is observed in clinical presentations and disease course across patients [4]. Additionally, molecular mechanisms such as oxidative stress and defective detoxification pathways, together with genetic variations in enzymes (glutathione S-transferases), may influence inflammatory responses [7].

Advances in understanding psoriasis immune pathways have led to the development of targeted biologic therapies directed against key inflammatory cytokines, including tumor necrosis factor (TNF)-α, interleukin (IL)-17, IL-12/23, and IL-23 [8].

These therapies revolutionized expected clinical outcomes, with a high proportion of patients achieving Psoriasis Area and Severity Index of 90 (PASI 90) or PASI100 responses in both clinical trials and real-world practice. Of these, IL-17 and IL-23 have demonstrated superior efficacy compared to earlier agents, establishing a new standard of care for moderate-to-severe psoriasis [9]. Differences in mechanisms of action between these classes are reflected in response kinetics, with IL-17 inhibitors associated with a more rapid onset of clinical improvement and IL-23 with sustained long-term effectiveness [10].

Early clinical response is increasingly recognized as a critical determinant of long-term treatment outcomes and therapeutic optimization. Achieving a rapid response enables early identification of responders versus non-responders, allowing timely treatment adjustments, minimizing exposure to ineffective therapies, and improving overall disease control.

Despite these therapeutic advances, treatment response remains heterogeneous, with interindividual variability in the speed and magnitude of clinical outcomes [11].

Real-world data from Central and Eastern Europe have shown that although biologic therapies lead to substantial reductions in disease severity, a subset of patients do not achieve optimal disease control [1,11]. Differences in drug survival across biologic classes also highlight the variability in long-term effectiveness and treatment persistence [9]. These observations underscore the need for improved strategies to predict therapeutic response.

To address these challenges of individual variability and the need for individualized therapeutic approaches, pharmacogenetics has emerged as a promising tool [12]. Genetic variants may influence both disease susceptibility and treatment response by modulating key inflammatory pathways. It has been shown that genetic variation contributes to interindividual differences in response to biologic therapy, supporting the potential role of pharmacogenetic profiling in personalized medicine [13].

Among the candidate genes, *IL12B* is a relevant target given its central role in psoriasis pathogenesis. The *IL12B* gene encodes for the p40 subunit shared by IL-12 and IL-23, both cytokines involved in the differentiation and activation of Th1 and Th17 cells [14]. Large-scale genetic association studies have consistently identified *IL12B* as a psoriasis susceptibility locus, with genome-wide significant associations reported in European populations [4]. These findings have been validated in independent cohorts, confirming the role of *IL12B* as a key genetic determinant in psoriasis pathogenesis [15].

In addition to its role in disease susceptibility, pharmacogenetic studies suggest that *IL12B* SNPs may also influence the biologic therapy response. In a prospective real-world cohort, the *IL12B* rs3213094 polymorphism was associated with differential responses to biologic therapy, with CT carriers exhibiting greater improvement in PASI scores at week 12 compared with CC homozygotes, particularly among patients treated with ustekinumab, with genetic variation correlating with longitudinal improvements in PASI [13,16]. Other pharmacogenetic studies have identified *IL12B* as a candidate gene associated with variability in treatment response alongside *TNFAIP3* and *CD84*, both of which encode immunoregulatory proteins [16]. Variants like rs3213094 and rs3212220 have also been associated with psoriasis susceptibility in different populations. However, the available evidence remains heterogeneous, with differences in study design, patient populations, biologic treatments, and outcome definitions limiting the consistency and clinical applicability of current data [13]. Importantly, allele-specific differences may translate into functional variability in cytokine signaling, potentially influencing both the magnitude and the speed of response to biologic therapies.

Polymorphisms in *IL23R*, which encodes the IL-23 receptor, have also been strongly associated with psoriasis susceptibility across multiple populations. Variants such as rs11209026 (Arg381Gln) confer protection against psoriasis and impair IL-23-induced Th17 effector responses [17,18]. Prior work from our group demonstrated that carriers of the A allele (AA + GA) of rs11209026 have significantly reduced odds of developing psoriasis compared with GG homozygotes, supporting the protective role of this variant [19]. However, *IL23R* polymorphisms have not shown consistent associations with biologic treatment response, so their role as predictors remains undefined [16,20].

Most genetic and pharmacogenetic studies in psoriasis have been conducted in Western European or Asian populations, while data from Central and especially Eastern Europe remain limited [3]. Differences in genetic background, healthcare infrastructure, protocols, and access to biologic therapies may contribute to regional variations in prevalence, disease burden, and treatment outcomes, highlighting the need for research in these understudied populations.

Accordingly, this study aimed to assess the association between *IL12B* rs3213094 and *IL23R* rs11209026 SNPs and treatment response to biologic therapy in a real-world cohort of Romanian patients with moderate-to-severe psoriasis, while also evaluating longitudinal PASI and Dermatology Life Quality Index (DLQI) dynamics and identifying independent predictors of early therapeutic response.

## 2. Materials and Methods

### 2.1. Study Design and Population

This multicenter observational study was conducted in Cluj-Napoca, Romania, following approval by the Ethics Committee on 7 July 2025. A total of 92 patients with psoriasis were recruited from the Department of Dermatology at the Emergency County Hospital Cluj and a private dermatology practice in Cluj-Napoca. Consecutive patients meeting the inclusion criteria were enrolled for genetic sampling between August 2025 and February 2026. Clinical response data at weeks 12, 24, 36, and 48 were retrospectively extracted from medical records, as all included patients had already completed routine follow-up evaluations before study inclusion. The present pharmacogenetic response cohort partially overlapped with our previously published Romanian psoriasis susceptibility cohort from the same participating centers and enrollment period [19], although the current study specifically included patients with available longitudinal biologic treatment-response data.

### 2.2. Inclusion and Exclusion Criteria

All patients included were ≥18 years of age and diagnosed with moderate-to-severe psoriasis vulgaris requiring systemic therapy, defined as body surface area (BSA) >10%, involvement of special areas, or inadequate response to topical therapy, in accordance with International Psoriasis Council consensus criteria [21]. Diagnosis was established clinically and, in atypical cases, confirmed by histopathological examination by the attending dermatologist. At the time of inclusion, all patients were treated with biologic therapy (anti-IL-17A, anti-IL-23, or anti-TNF-α monoclonal antibodies), representing their first biologic treatment, with no prior exposure to biologic agents.

Exclusion criteria included patients with other forms of psoriasis, those with other autoimmune diseases such as inflammatory bowel diseases and ankylosing spondylitis, given the shared genetic susceptibility and involvement of the IL-23/Th17 pathway across these immune-mediated disorders [22,23], and those with active malignancy. Patients treated with agents represented by small therapeutic subgroups (anti-IL17A/F and anti-IL12/23 inhibitors) were also excluded to ensure statistical robustness. Although anti-IL-12/23 therapy is mechanistically relevant to *IL12B* signaling, the number of patients receiving Ustekinumab across participating centers during the study period was insufficient to allow statistically meaningful subgroup analyses. Inclusion of a very limited anti-IL-12/23 cohort could therefore have reduced the robustness and interpretability of intergroup comparisons.

### 2.3. Ethics Approval and Consent to Participate

The study was conducted in accordance with the Declaration of Helsinki. Written informed consent was obtained from all participants before inclusion. The study protocol was approved by the Ethics Committee of the “Iuliu Haţieganu” University of Medicine and Pharmacy, Cluj-Napoca (Approval No. AVZ 198, issued on 7 July 2025).

### 2.4. Clinical and Demographic Data Collection

Demographic and clinical data collected included age, sex, dyslipidemia, body mass index (BMI), hypertension, diabetes mellitus, cardiovascular disease (including atherosclerosis, or history of major cardiovascular events such as myocardial infarction, ischemic stroke, and documented coronary artery disease—including angina or coronary revascularization procedures), psoriatic arthritis (diagnosis based on rheumatologist’s evaluation), and nail involvement.

BMI was calculated as weight (kg) divided by height squared (m^2^), and patients were divided into normal weight, overweight, and obesity grades I–III [24]. Height and weight were measured by a trained nurse using standardized procedures. Body weight was assessed using a calibrated electronic medical scale (Seca GmbH & Co. KG, Hamburg, Germany), and height was measured with a fixed wall-mounted stadiometer (SECA 206, Seca GmbH & Co. KG, Hamburg, Germany). Dyslipidemia was categorized as hypercholesterolemia (total cholesterol > 200 mg/dL), hypertriglyceridemia (serum triglyceride > 150 mg/dL), or mixed dyslipidemia according to contemporary ESC/EAS guidelines [24]. Participants who did not meet these criteria were categorized as having no dyslipidemia. Laboratory parameters used to define dyslipidemia were extracted from patients’ medical records before initiation of biologic therapy and, where applicable, before initiation of lipid-lowering treatment.

The efficacy of treatment was evaluated using PASI at baseline and at weeks 12, 24, 36, and 48. Early responders were defined as patients achieving PASI75 (≥75% reduction from baseline PASI) at week 12. To assess response kinetics, percentage PASI improvement was calculated at each time point relative to baseline. Additionally, the impact of disease on quality of life was assessed using the DLQI score at the same time points.

### 2.5. Polymorphisms Studied

We selected two SNPs, rs3213094 in the *IL12B* gene and rs11209026 in the *IL23R* gene, based on their previously reported involvement in psoriasis susceptibility and treatment response.

*IL12B* encodes the p40 subunit shared by interleukin-12 and interleukin-23, playing a central role in the IL-23/Th17 inflammatory pathway, which is a key driver of psoriasis pathogenesis and a major therapeutic target of biologic agents [25]. Genetic variants within the *IL12B* locus, including SNPs such as rs3212227, rs6887695, and rs2546890, have been consistently associated with psoriasis susceptibility and other immune-mediated diseases [26,27,28], and several pharmacogenetic studies have suggested that *IL12B* variants may influence response to biologic therapies, particularly anti-TNF agents [28,29]. However, these studies have primarily focused on other *IL12B* polymorphisms, while data regarding rs3213094 remain scarce. Therefore, the present study investigates a less-explored variant within the *IL12B* locus, addressing a gap in the current pharmacogenetic literature.

Similarly, polymorphisms in *IL23R*, including rs11209026, and other variants such as rs7530511, have been associated with psoriasis susceptibility across different populations [27] and may also contribute to variability in treatment response. However, current evidence remains limited and heterogeneous [30].

### 2.6. Sample Collection, DNA Isolation, and Genotyping

Peripheral blood was collected from the cubital vein into EDTA tubes under sterile conditions. Genomic DNA was isolated from whole blood using the PureLink Genomic DNA Mini Kit (Thermo Fisher Scientific, Van Allen Way, Carlsbad, CA, USA), as previously described in our companion susceptibility study [19]. DNA concentration and purity were assessed using a NanoDrop™ spectrophotometer (Thermo Fisher Scientific, Carlsbad, CA, USA). Genotyping of *IL12B* rs3213094 and *IL23R* rs11209026 was performed using predesigned TaqMan SNP Genotyping Assays (assay IDs: C_29927086_10 and C_1272298_10; Applied Biosystems, Waltham, MA, USA) on a QuantStudio 3 Real-Time PCR System (Thermo Fisher Scientific, Carlsbad, CA, USA), according to the manufacturer’s protocol. Approximately 5–10% of samples were randomly genotyped in duplicate for quality control, yielding a concordance rate >99%. Negative controls were included in each run to monitor contamination, and genotype calling was performed using TaqMan Genotyper Software v1.6 (Applied Biosystems, Carlsbad, CA, USA).

### 2.7. Statistical Analysis

Continuous variables are summarized as median (interquartile range), and categorical variables as number (percentage). Comparisons of continuous variables between groups were performed using the Mann–Whitney U test, while categorical variables were analyzed using the χ^2^ test or Fisher’s exact test, as appropriate.

To evaluate longitudinal PASI response kinetics, repeated-measures analysis of variance (ANOVA) was used to test the effect of time on percentage PASI improvement (after logarithmic transformation), with Greenhouse–Geisser correction applied where appropriate. Genotype-by-time interaction terms were assessed to evaluate whether response trajectories differed by genotype.

For categorical early response, the association between *IL12B* rs3213094 genotype and PASI75 achievement at week 12 was evaluated using χ^2^ or Fisher’s exact test, as appropriate. Hardy–Weinberg equilibrium (HWE) was assessed for each polymorphism using the χ^2^ test. Because of the low frequency of the *IL23R* rs11209026 minor allele, HWE interpretation should be interpreted cautiously.

A multivariable logistic regression model was constructed to assess whether *IL12B* rs3213094 independently predicted PASI75 at week 12. Variables with *p* < 0.1 in univariate analysis were entered into the multivariable model. Clinically relevant covariates, including age, sex, body mass index (BMI), psoriatic arthritis, and dyslipidemia, were evaluated in univariate analysis but were not retained in the final model because they did not meet the predefined inclusion threshold. Results are reported as odds ratios (ORs) with 95% confidence intervals (CIs).

Changes in DLQI over time were analyzed similarly using repeated-measures ANOVA, including genotype-by-time interaction terms.

All tests were two-sided, and *p* ≤ 0.05 was considered statistically significant. All statistical analyses were performed using MedCalc^®^ Statistical Software version 23.4.9 (MedCalc Software Ltd., Ostend, Belgium; https://www.medcalc.org; 2026).

Because of the exploratory nature of the study and the limited sample size, no formal correction for multiple comparisons was applied. The findings, especially those from subgroup analyses, should therefore be interpreted cautiously and validated in larger cohorts.

## 3. Results

### 3.1. Study Population

A total of 92 Romanian patients with moderate-to-severe psoriasis vulgaris were included in the present study. The *IL12B* rs3213094 CC and *IL23R* rs11209026 GG genotypes were the most frequently identified. Genotype distributions for *IL12B* rs3213094 were consistent with HWE. Interpretation of HWE testing for *IL23R* rs11209026 was limited by the low frequency of the minor allele. Anti-IL-17 agents represented the most frequently prescribed therapeutic class. A marked and progressive reduction in disease severity was observed over time, with substantial clinical improvement achieved at week 12, further improvement at week 24, and sustained near-complete responses by weeks 36 and 48. Baseline demographic and clinical characteristics of the study population, alongside genotype distribution, treatment classes, and longitudinal PASI changes, are summarized in Table 1.

A significant reduction in PASI scores over time was observed. Repeated-measures ANOVA demonstrated a statistically significant effect of time on percentage PASI improvement (*p* < 0.001), indicating a robust overall treatment effect.

### 3.2. IL12B rs3213094 and PASI Improvement

The interaction between time and genotype was analyzed to determine whether the genetic background influenced treatment response dynamics. A significant interaction between time and *IL12B* rs3213094 genotype was identified (*p* = 0.03), indicating differences in treatment response kinetics according to genotype. Conversely, no significant interaction between time and *IL23R* rs11209026 was observed (*p* = 0.82).

At week 12, T allele carriers demonstrated a higher median percentage PASI improvement compared with CC homozygotes in *IL12B* (90% vs. 65.7%). In contrast, no significant genotype-related differences were observed at weeks 24, 36, or 48, indicating that early differences in treatment response attenuated over time (Table 2).

Approximately half of the patients achieved PASI75 after 12 weeks of treatment (47 patients (51%)). Several variables were tested to evaluate their association with PASI75 at 12 weeks (Table 3). A trend toward differences among biologic classes was observed (*p* = 0.073), while *IL12B* rs3213094 genotype was significantly associated with PASI75 achievement in univariate analysis (*p* = 0.031).

To determine which variables were independently associated with treatment response, a multivariate logistic regression analysis was performed with PASI75 achievement at week 12 as the dependent variable. Variables with a *p* < 0.1 in univariate analysis were included in the model. Clinically relevant covariates, including age, sex, BMI, psoriatic arthritis, and dyslipidemia, were evaluated in univariate analysis, but did not meet the predefined criteria for inclusion and were not introduced in the multivariable model. The dominant genetic model for *IL12B* rs3213094 (CT + TT vs. CC) and the use of anti–IL-17A therapy were independently associated with PASI75 achievement (Table 4).

No significant differences in biologic class distribution were identified according to IL12B rs3213094 genotype (*p* = 0.190).

We evaluated a potential confounding situation regarding the treatment. Thus, exploratory subgroup analyses according to biologic class were performed. Higher PASI75 rates among *IL12B* rs3213094 CT + TT carriers were noted in all biologic classes, but statistical significance was reached only in the anti-IL-17 subgroup (Table 5).

### 3.3. DLQI Evolution

DLQI scores significantly improved over time, reflecting a progressive reduction in disease burden during follow-up (*p* < 0.001). No significant interaction between time and *IL12B* rs3213094 genotype was identified (*p* = 0.212), indicating that genotype did not influence the dynamics of quality-of-life improvement. Similarly, no significant interaction between time and *IL23R* rs11209026 genotype was observed (*p* = 0.821). Median DLQI values at each time point according to IL12B genotype are presented in Table 6.

## 4. Discussion

### 4.1. IL12B Polymorphism and Early Biologic Response in Psoriasis

In this real-world study of Romanian patients with moderate-to-severe psoriasis treated with biologic therapy for the first time, we investigated whether selected SNPs in *IL12B* and *IL23R* influence clinical response. T-allele carriers in *IL12B* rs3213094 showed a significantly greater percentage of PASI improvements at week 12 compared with CC homozygotes. This difference was limited to the early stages of treatment and attenuated over time (24, 36, and 48 weeks). In contrast, no significant association was identified for *IL23R* rs11209026. These findings suggest that *IL12B* genetic variation may play an important role in early response rather than in long-term efficacy outcomes [20,31].

Previous studies investigated the role of *IL12B* SNPs in modulating response to biologic therapy in psoriasis. In a large Dutch real-world cohort, *IL12B* polymorphisms, including rs3213094, were associated with early PASI reduction in patients treated with Ustekinumab, especially when assessed using continuous ΔPASI measures. For dichotomous endpoints such as PASI75, the association was less evident, suggesting that *IL-12B* variants may influence the magnitude of the early response [16]. Notably, most previously reported associations were observed in cohorts treated with agents targeting the IL-12/23 axis. In contrast, anti-IL-12/23 therapies were not included in the present cohort because of the limited number of eligible patients. Nevertheless, the association between *IL12B* rs3213094 and early treatment response remained detectable in a population treated predominantly with anti–IL-17 and anti-TNF agents, suggesting that the influence of *IL12B* genetic variation may extend beyond responsiveness to ustekinumab alone and reflect a broader immunogenetic effect on treatment response pathways in psoriasis.

Because therapeutic class was independently associated with PASI75 achievement in the multivariable model, a potential treatment-related confounding effect was further explored through subgroup analyses stratified by biologic class. Higher PASI75 response rates among *IL12B* rs3213094 CT + TT carriers were observed across all biologic classes; however, statistical significance was reached only in the anti-IL-17 subgroup. These findings suggest that the overall association between *IL12B* rs3213094 and early treatment response may be driven predominantly by patients receiving IL-17 inhibitors. Nevertheless, the consistent numerical trend across biologic classes may support a broader contribution of IL12B-related pathways to biologic responsiveness in psoriasis.

Several other studies have explored the role of the *IL12B* gene in response to anti-TNF-α therapy, although the SNPs investigated differed across studies. In a Spanish cohort, *IL12B* polymorphisms were associated with PASI75 achievement at 3 and 6 months under anti-TNF-α inhibitors [29]. Additionally, pharmacogenetic data identified other *IL12B* SNPs associated with PASI75 achievement with Adalimumab and Infliximab [28], suggesting that the association is not limited to rs3213094 in *IL12B* but rather reflects variation within the *IL12B* locus. Interestingly, in our previously published susceptibility analysis performed in a related Romanian cohort, *IL12B* rs3213094 was not associated with psoriasis susceptibility [19]. In contrast, the present study identified an association between this variant and early biologic treatment response. These findings suggest that *IL12B* rs3213094 may not substantially influence disease susceptibility itself, but rather modulate immunologic pathways involved in treatment responsiveness and early inflammatory resolution.

Furthermore, systematic reviews have reported different SNPs in *IL12B* as potential biomarkers of response to biologic therapy [30,32], but the heterogeneity across the included studies reflects differences in outcome definitions. In contrast, our study demonstrates a significant association between *IL12B* rs3213094 and early treatment response—at 12 weeks—defined as both continuous PASI improvement and a PASI75.

### 4.2. Biological Context of IL12B in Early Response

*IL12B* encodes the p40 shared subunit of IL-12 and IL-23. Thus, its role in treatment response is biologically justified, as variations at this locus could influence the intensity of cytokine-orchestrated inflammation at the start of therapy.

Early clinical response may suppress hyperactive immune signaling along the IL-23/Th17 axis [15,33]. In this context, *IL12B* SNPs may preferentially influence early PASI reduction rather than long-term reduction, which is generally achieved across most biologic agent classes.

Accordingly, *IL12B* polymorphisms may have a more pronounced effect on early PASI reduction than on long-term outcomes, which tend to converge across treatment groups once inflammation is adequately controlled. This hypothesis is consistent with our findings, where genotype-related differences were evident at week 12 but diminished over time, suggesting a temporal effect of genetic variation on treatment response [31].

### 4.3. Early Response Kinetics and Attenuation of the Genetic Effect over Time

In phase 3 trials, Secukinumab achieved PASI75 rates over 75% by week 12. Similarly, head-to-head comparisons between Ixekizumab and Guselkumab revealed significantly faster achievement of PASI90 and PASI100 in the Ixekizumab-treated cohort during the early treatment phase [34,35]. These data align with our multivariate analysis, in which IL-17A blockade was independently associated with PASI75 achievement at week 12. Beyond clinical improvement, molecular studies have shown that IL-17 antagonism rapidly reduces inflammatory gene expression, particularly within the first 12 weeks of treatment [36].

In our cohort, the association between *IL12B* rs3213094 and treatment response was limited to week 12 and attenuated at later follow-up visits. As treatment progresses and inflammatory activity declines, clinical outcomes tend to converge across patients, which may reflect a ceiling effect, reducing the ability to detect genotype-related differences at later time points [31].

### 4.4. Lack of Association for IL23R rs11209026

In contrast to *IL12B* rs3213094, no significant association was observed between *IL23R* rs11209026 and treatment response in our cohort. Although several meta-analyses and genetic studies reported *IL23R* SNPs associated with psoriasis susceptibility [19,26,33], their role as predictive biomarkers of response to biologic therapy remains undefined. There is a substantial heterogeneity in cohort design and outcome definitions across studies evaluating *IL23R* polymorphisms [31]. Thus, the absence of association in our work may reflect limited statistical power due to the low frequency of the minor allele and may also suggest that genetic determinants of disease susceptibility are not necessarily predictors of treatment response.

### 4.5. Discrepancy Between Clinical Response and Quality of Life Index (DLQI)

The present study demonstrated a significant, progressive improvement in DLQI scores over the follow-up period, reflecting a substantial reduction in disease burden with biologic therapy. These findings are consistent with previous evidence showing that biologic therapies lead to marked improvements not only in clinical severity but also in patient-reported outcomes and quality of life [37]. At baseline, patients had higher DLQI scores, highlighting the well-known negative impact of psoriasis on daily functioning, psychological well-being, and social interactions. Psoriasis is associated with psychosocial burden, including stigma, reduced self-esteem, and impaired social functioning, all of which contribute significantly to diminished quality of life [38].

Following treatment initiation, DLQI scores decreased rapidly, with clinically meaningful improvement observed early on during follow-up. The early improvement observed in our cohort aligns with evidence showing that rapid clinical response—particularly with IL-17 inhibitors—is closely associated with early gains in quality of life [39]. Furthermore, head-to-head clinical trials have demonstrated that faster skin clearance translates into more days without psoriasis impacting daily life, reinforcing the clinical relevance of early therapeutic response [35].

In randomized clinical trials, a substantial proportion of patients achieve DLQI scores of 0 or 1—indicating no impact of psoriasis on daily life—with rates over 60% under highly effective biologic therapies [38]. Similarly, real-world data show that approximately one-third of patients maintain DLQI 0/1 responses at one year, particularly among those receiving IL-17-targeted therapies [40].

Despite these overall improvements, no significant interaction between *IL12B* rs3213094 genotype and DLQI evolution was observed in our cohort. Although carriers of the T allele demonstrated a superior early clinical response, this advantage did not translate into measurable differences in quality-of-life outcomes. This apparent discrepancy is supported by emerging evidence indicating that improvements in clinical severity do not always correlate directly with patient-reported outcomes. In a large prospective cohort, only one-third of patients achieving PASI75 also reached optimal DLQI improvement, highlighting a discordance between objective and subjective treatment responses [41]. This divergence likely reflects the multidimensional nature of DLQI, which is influenced not only by clinical severity but also by psychological and social factors. Consequently, genetic predictors such as *IL12B* rs3213094 may primarily impact objective inflammatory pathways, with limited influence on patient-perceived disease burden.

### 4.6. Strengths and Limitations

This study has several important strengths.

First, it reflects a real-world cohort of patients with moderate-to-severe psoriasis vulgaris, increasing the applicability of the results to real-world clinical settings. Furthermore, the longitudinal design with multiple follow-up timepoints allowed for a dynamic assessment of treatment response, capturing both early and sustained long-term effects of biologic therapy. Finally, integrating objective clinical outcomes (PASI) and patient-reported outcomes (DLQI) provides a comprehensive evaluation of treatment effectiveness from both physician and patient perspectives.

Another key strength is the focus on pharmacogenetics, specifically the investigation of *IL12B* rs3213094, a variant located within a biologically relevant pathway involved in psoriasis pathogenesis. This adds novel data to the limited body of evidence on genetic predictors of treatment response. Importantly, this study addresses a significant gap in the current literature, as populations from Eastern Europe, including Romanian patients, remain underrepresented in pharmacogenetic and clinical studies of psoriasis. Furthermore, the use of multivariable regression analysis strengthens the robustness of the findings by accounting for potential confounding factors.

However, several limitations should be acknowledged.

First, the observational design of the study does not allow for causal inference and may be subject to residual confounding despite adjustment. Second, the relatively modest sample size may have limited statistical power, particularly for genetic analyses and subgroup comparisons. This limitation was especially relevant for the *IL23R* rs11209026 polymorphism, for which only seven patients carried the minor allele, substantially reducing the ability to detect significant genotype–treatment associations and likely contributing to the null findings observed for this variant. Similarly, patients receiving anti-IL-12/23 therapy were not included because the number of eligible cases was insufficient to allow statistically reliable subgroup analyses, despite the biological relevance of IL12B signaling in this therapeutic pathway. Moreover, the exploratory subgroup analyses stratified by biologic class included relatively small patient numbers after stratification, which may have limited the ability to detect statistically significant genotype-specific effects across all treatment categories. In addition, the evaluation of multiple SNPs, clinical endpoints, and longitudinal timepoints may have increased the risk of type I error, and the observed associations should therefore be interpreted cautiously. Third, the study population comprised patients with moderate-to-severe psoriasis who initiated biologic therapy at tertiary dermatology centers. While this represents a clinically relevant population, it may limit the generalizability of the findings to patients with milder disease or those treated in other clinical settings. Additionally, the relative ethnic homogeneity of the study population may restrict the extrapolation of these results to other populations with different genetic backgrounds.

Finally, although both clinical and patient-reported outcomes were assessed, more detailed phenotypic stratification and the inclusion of additional molecular or inflammatory biomarkers could have provided further insight into genotype–phenotype relationships and the underlying mechanisms of treatment response.

## 5. Conclusions

In this real-world cohort of Romanian patients diagnosed with moderate-to-severe psoriasis vulgaris, the *IL12B* rs3213094 polymorphism was independently associated with early treatment response to biologic therapy. Carriers of the T allele had a significantly higher probability of achieving PASI75 at week 12 (as compared to CC homozygote patients), highlighting the pharmacogenomic influence of the T allele on early treatment response dynamics. Furthermore, treatment with anti-IL-17A agents was independently associated with earlier and better clinical response, supporting the efficacy of this class of agents in disease control in a real-world setting. Taken together, our findings suggest that *IL12B* rs3213094 could be a potential biomarker for predicting which psoriasis patients will respond earlier to biologic drugs. To our knowledge, this is the first study evaluating this polymorphism in patients with psoriasis receiving their first biologic therapy in Romania. We encourage further investigation of these associations in a larger study population to validate their clinical utility in personalized treatment strategies.

## Figures and Tables

**Table 1:** Baseline Characteristics, Genotype Distribution, Therapeutic Classes, and PASI Evolution (*N* = 92).

Variable		Value
Age, years		49 (39.25–59.00)
Male sex, *n* (%)		59 (64.1%)
BMI, kg/m^2^		28.19 (24.44–31.62)
Hypertension, *n* (%)		32 (34.8%)
Diabetes mellitus, *n* (%)		21 (22.8%)
Cardiovascular disease, *n* (%)		12 (13.0%)
Dyslipidemia, *n* (%)		57 (62.0%)
Psoriatic arthritis, *n* (%)		27 (29.3%)
Nail involvement, *n* (%)		45 (48.9%)
*IL12B* rs3213094	CC	65 (70.7%)
	CT	22 (23.9%)
	TT	5 (5.4%)
	CT + TT	27 (29.3%)
*IL23R* rs11209026	GG	85 (92.4%)
	GA	5 (5.4%)
	AA	2 (2.2%)
	GA + AA	7 (7.6%)
Therapeutic class—Anti-TNF		33 (35.9%)
Therapeutic class—Anti-IL17		43 (46.7%)
Therapeutic class—Anti-IL23		16 (17.4%)
Baseline PASI (absolute value)		20.65 (14.00–25.75)
PASI reduction at 12 weeks (%)		76.63 (51.71–95.63)
PASI reduction at 24 weeks (%)		97.89 (83.46–100.00)
PASI reduction at 36 weeks (%)		100.00 (91.67–100.00)
PASI reduction at 48 weeks (%)		100.00 (91.41–100.00)

**Table 2:** Percentage PASI Improvement According to *IL12B* rs3213094 Genotype.

Timepoint	CC	CT + TT	*p*-Value
Week 12	65.7% (33.2–95.3)	90.0% (77.5–100)	**0.003**
Week 24	95.4% (82.9–100)	100% (90–100)	0.262
Week 36	98.8% (91.7–100)	100% (94.6–100)	0.317
Week 48	100% (91.0–100)	100% (91.5–100)	0.846

Bold values indicate statistically significant results (*p* < 0.05).

**Table 3:** PASI75 Achievement at Week 12.

Variable		PASI75 Achieved *n* (%)	PASI75 Not Achieved *n* (%)	*p*-Value
Age		49 (38; 59)	50 (40; 61)	0.642 *
Sex—Male		33 (51.7%)	26 (48.3%)	0.821
Sex—Female		14 (42.4%)	19 (57.6%)	
BMI		28.2 (24.2; 31.5)	28.1 (24.6; 31.9)	0.867 *
Hypertension		17 (53.1%)	15 (46.9%)	0.925
Diabetes mellitus		10 (47.6%)	11 (52.4%)	0.643
Cardiovascular disease		8 (66.7%)	4 (33.3%)	0.247
Dyslipidemia		29 (61.7%)	29 (62.2%)	1
Psoriatic arthritis		14 (51.9%)	13 (48.1%)	0.925
Nail involvement		23 (51.1%)	22 (48.9%)	0.996
Therapeutic class				
Anti-TNF		12 (36.4%)	21 (63.6%)	**0.073**
Anti-IL17		27 (62.8%)	16 (37.2%)	
Anti-IL23		8 (50.0%)	8 (50.0%)	
*IL12B* rs3213094	CC	28 (43.1%)	37 (56.9%)	**0.031**
	CT + TT	19 (70.4%)	8 (29.6%)	

Bold values indicate statistically significant results (*p* < 0.05); * Mann–Whitney test.

**Table 4:** Multivariate Logistic Regression Analysis for PASI75 Achievement at Week 12.

Variables	B	*p*	OR	95% CI for OR	
				Min	Max
*IL12B* rs3213094 CT + TT	1.455	**0.007**	4.285	1.500	12.239
**Therapeutic class**		**0.029**			
Anti-TNF-α (reference)	-	-	1.000	-	-
Anti-IL-17A	1.373	**0.009**	3.946	1.416	10.998
Anti-IL–23	0.466	0.478	1.593	0.440	5.770
Constant	0.246	0.352	1.279		

Bold values indicate statistically significant results (*p* < 0.05).

**Table 5:** Exploratory Subgroup Analyses of PASI75 Achievement at Week 12 According to Biologic Class and *IL12B* rs3213094 Genotype.

Therapeutic Class	*IL12B* rs3213094	PASI75 Achieved *n* (%)	PASI75 Not Achieved *n* (%)	*p*
Anti-TNF	CC	7 (31.8)	15 (68.2)	0.470
	CT + TT	5 (45.5)	6 (54.5)	
Anti-IL17	CC	18 (52.9)	16 (47.1)	**0.016**
	CT + TT	9 (100)	0 (0)	
Anti-IL23	CC	3 (33.3)	6 (66.7)	0.315
	CT + TT	5 (71.4)	2 (28.6)	

Bold values indicate statistically significant results (*p* < 0.05).

**Table 6:** DLQI Evolution According to IL12B rs3213094 Genotype.

Timepoint	CC (Median)	CT + TT (Median)	*p*-Value
Baseline	21	18	0.227
Week 12	6	6	0.150
Week 24	2	2	0.735
Week 36	1	0	0.960
Week 48	2	2	0.543

## Data Availability

The data presented in this study are available on request from the corresponding author due to privacy concerns.

## Abbreviations

The following abbreviations are used in this manuscript:

SNP	Single Nucleotide Polymorphism
PASI	Psoriasis Area and Severity Index
TNF-α	Tumor Necrosis Factor-α
DLQI	Dermatology Life Quality Index
GWAS	Genome-Wide Association Study
HWE	Hardy–Weinberg equilibrium
NF-κB	Nuclear Factor kappa-light-chain-enhancer of activated B cells
HLA	Human Leukocyte Antigen
TNFAIP3	Tumor Necrosis Factor Alpha-Induced Protein 3
CD	Cluster of Differentiation
BSA	Body Surface Area
BMI	Body Mass Index
ESC/EAS	European Society of Cardiology/European Atherosclerosis Society
